# Estimating the Risk of Influenza-Like Illness Transmission Through Social Contacts: Web-Based Participatory Cohort Study

**DOI:** 10.2196/publichealth.8874

**Published:** 2018-04-09

**Authors:** Ta-Chien Chan, Tsuey-Hwa Hu, Jing-Shiang Hwang

**Affiliations:** ^1^ Research Center for Humanities and Social Sciences Academia Sinica Taipei City Taiwan; ^2^ Institute of Statistical Science Academia Sinica Taipei City Taiwan

**Keywords:** flu transmission, social networks, contact diary, diet, exercise, sleep quality

## Abstract

**Background:**

Epidemiological studies on influenza have focused mostly on enhancing vaccination coverage or promoting personal hygiene behavior. Few studies have investigated potential effects of personal health behaviors and social contacts on the risk of getting influenza-like illness (ILI).

**Objective:**

Taking advantage of an online participatory cohort, this study aimed to estimate the increased risk of getting ILI after contact with infected persons and examine how personal health behaviors, weather, and air pollution affect the probability of getting ILI.

**Methods:**

A Web-based platform was designed for participants to record daily health behaviors and social contacts during the influenza season of October 1, 2015 to March 31, 2016, in Taiwan. Data on sleep, diet, physical activity, self-reported ILI, and contact with infected persons were retrieved from the diaries. Measurements of weather and air pollutants were used for calculating environmental exposure levels for the participants. We fitted a mixed-effects logistic regression model to the daily measurements of the diary keepers to estimate the effects of these variables on the risk of getting ILI.

**Results:**

During the influenza season, 160 participants provided 14,317 health diaries and recorded 124,222 face-to-face contacts. The model estimated odds ratio of getting ILI was 1.87 (95% CI 1.40-2.50) when a person had contact with others having ILI in the previous 3 days. Longer duration of physical exercise and eating more fruits, beans, and dairy products were associated with lower risk of getting ILI. However, staying up late was linked to an elevated risk of getting ILI. Higher variation of ambient temperature and worse air quality were associated with increased risk of developing ILI.

**Conclusions:**

Developing a healthier lifestyle, avoiding contact with persons having ILI symptoms, and staying alert with respect to temperature changes and air quality can reduce the risk of getting ILI.

## Introduction

### Background

Seasonal influenza epidemics cause a high disease burden, including direct costs of health services and households and indirect costs due to productivity losses worldwide every year [1-3]. Preventing influenza transmission includes pharmaceutical approaches [4,5] and nonpharmaceutical approaches [6]. Human face-to-face contact is the major transmission route for influenza, and infection probability will be elevated with close contact [7,8]. The household is one of the places where people have close contact and is also the bridge of influenza transmission between schools and the community [9]. If one member of a household gets influenza, the risk of infection in a household contact can be up to 38% [10]. Although social contact enhances the risk of influenza infection, there are still some ways to break down this transmission route, including personal prevention and avoiding social contact. In personal prevention, enhancing personal immunity through physical exercise and keeping good hygiene behaviors such as hand washing and wearing a face mask are effective approaches to reduce the risk of contracting an influenza infection [11]. Avoiding face-to-face contact with infected people should also reduce the chance of getting an infection. However, it has been a challenge to prospectively quantify how social contact affects the chance of influenza transmission, including the number of instances, intensity, and duration of contacts [8]. In one social network study, the researchers found that people at the center of a network got the flu earlier, and that information could be used for early detection of influenza epidemics [12]. This implies that people with more social ties might have higher chances of getting infection or spreading the virus to others. In addition to those risk factors from human beings, external weather conditions not only facilitate influenza transmission but also affect people’s immunity [13,14]. Recently, one study reported that increased exposure to ambient fine particulate matter (PM_2.5_) also contributed to 10.7% of incident influenza cases in China [15]. From observation of human cell lines, exposure to ozone also is associated with increased influenza susceptibility [16].

### Objectives

In this study, we retrieved data from an online diary platform called ClickDiary [17], which provided participants a simple way to prospectively record daily health behaviors, influenza symptoms in themselves, and their contact persons during an influenza season in Taiwan. There have previously been some studies using self-reporting of influenza-like illness (ILI) by volunteers to monitor influenza activity in the communities, such as the *Flutracking* platform in Australia [18] and *Flu Near You* in the United States and Canada [19], both of which are simple internet-based surveys to record the weekly ILI symptoms of the participants. However, the main purpose of those studies was to detect earlier aberration signals to detect flu epidemic. In contrast, with the comprehensive diary data, the purpose of this study was to estimate the increased risk of getting ILI symptoms after contact with infected persons and examine how personal health behaviors, weather, and air pollution affect the chance of getting ILI.

## Methods

### Ethics

This study was approved by the Institutional Review Board on Humanities and Social Science Research, Academia Sinica (AS-IRB-HS 02-13022). The diary data for analysis were stripped of personal identification information, which was replaced with a serial number to protect participants’ privacy.

### ClickDiary Program

We used a Web-based platform named “ClickDiary” [20] to collect data on participants’ daily health behaviors and social contact [17]. The participants, recruited from various channels, included university students, school teachers and administrative employees, volunteers at health promotion centers, hospital patients, and community college students, as well as other adults in the general population. This ClickDiary program started in May 2014 and ended in December 2016. Although 1432 participants contributed their diaries, active diary keepers numbered about 200 in any given week. The participants were required to click questionnaire options in the online diary at least twice a week. In the health diary, the participants recorded sleep duration and quality, mood, food intake, physical exercise, self-reported ILI and symptoms, and other information. The diet questionnaire is adopted and modified from an official diet questionnaire created by the Health Promotion Administration, Ministry of Health and Welfare, Taiwan. The exercise questionnaire is adopted from one health examination questionnaire [21]. The detailed diet and exercise questionnaires were listed in another published paper [17]. The dietary intake consisted of 14 major food items, and the participants were to report the number of portions (0, 0.5, 1, 2, 3, or 3+) consumed during the past 24 hours. Food items included vegetables, fruits, whole grains and roots, rice and flour, pork/beef/mutton, chicken/duck/goose, seafood, eggs, beans and pulses, dairy products, fried foods, processed foods, desserts, and sugary drinks. The participant reported whether he or she had ILI by selecting one of the 3 options, including none, probably, and definitely having ILI symptoms. The participants reporting ILI were also asked to select at least one of the specific symptoms, including fever (>38°C), cough, sneezing, chills, stuffy nose, sore throat, fatigue, diarrhea, and/or chest pain. In this study, we defined a participant as having ILI symptoms only when she or he selected “definitely having ILI symptoms.”

The contact diary was designed for collecting information on participants’ daily social contact. The definition of contact used was stricter than in other contact diary studies [22]. An instance of contact was defined as in-person exchange of at least three sentences [23].The participants recorded major contact attributes, including time and place of contact, who initiated it, the means of contact, duration, content, participant’s instrumental gain and mood, and the contact person’s location, mood, and ILI symptoms, if any. Each record in the contact diary represented a one-on-one contact. If the participant met many people at the same time, they recorded only the persons with whom they had exchanges of at least three sentences. A lottery-based reward system was designed to encourage the participants to fulfill the requirements. To ensure the quality of the data, we checked the data pattern of each participant every week. If a participant had not kept his or her diaries properly, the participant was put on an alert list, and any data entered by the participant would be excluded from the database for analysis.

The participants provided demographic information at sign-up, including age, gender, place of residence, marital status, and type of current job. The program also collected participants’ Big Five personality traits (openness, conscientiousness, extraversion, agreeableness, and neuroticism) [24], height and weight for calculating body mass index (kg/m^2^), perceived health status and happiness, the number (and characteristics) of people with whom they were in contact during the day, and a baseline health survey, which borrows items from the Taiwan Social Change Survey [25].

### Diary Data and Notation

We created a cohort of 160 participants who each recorded the two diaries at least 10 days a month for more than 2 months during the influenza season from October 1, 2015, to March 31, 2016. Some participants recorded their health diary most of the times, but their contact diary only occasionally, because it was more time-consuming. Although the participants were required to input in their diaries twice a week, we found that more than half of the participants in the cohort had recorded entries at least 3 times a week, and the average was 4 times a week. There are two major reasons why we did not include all data across the entire period. The first one is that our data showed that the overall self-reported ILI rate was low in the nonflu season. To focus on identifying the risk factors for ILI transmission, we restricted the studied period to the flu season. The other reason is that, for the first 2014-2015 flu season, we encountered a logistical problem for issuing the vouchers from mid-December 2014 to January 2015. This caused the participation rate to fall during that period, and the number of diaries was too small to conduct the analysis.

We introduce a notation to describe the variables for use in the statistical analysis in the following. When the *i*-th participant recorded the health diary for the *j*-th time on the day t_ij_ during the study period, we denoted Y_ij_=1 if the participant reported ILI symptoms and 0 otherwise. We then retrieved data logs of the participant’s health diary and contact diary for the 3 days before t_ij_. The binary variables Z_ij_=1 and C_ij_=1represent the participant self-reporting ILI and having contact with infected persons, respectively, during the past 3 days, and 0 otherwise. We count events in the previous 3 days based on the incubation period of influenza [10]. For each food item, the number of portions 3+ was coded as 4. The average number of portions of the *k*-th food category in the past 3 days is denoted by F_kij_. The participants’ selected daily exercise time from five categories ranging from none to 1-30, 31-60, 61-120, and 120+ minutes. We coded these 5 categories as 0, 15.5, 45.5, 90.5, and 120 min [26]. The average duration of exercise in the past 3 days is denoted by E_ij_. We denoted the average sleep time of the participants as S_ij_, and staying up after 1 AM at least once in the past 3 days as L_ij_.

### Weather and Air Pollution Data

To understand how environmental factors affect influenza transmission after controlling for health behaviors and social contacts, we included weather and air pollution data from ambient air quality monitoring stations maintained by Taiwan’s Environmental Protection Administration, excluding traffic, industrial, and background stations [27]. In this study, we selected 35 air monitoring stations closest to the townships the participants lived in. The measurements of weather and air pollutants at the selected stations were used for computing environmental exposure for the participants. We computed average values of daily mean temperature (°C), daily maximum 8-hour moving average concentration of O_3_ (ppm), daily mean concentration of PM_2.5_ (μg/m^3^) and other pollutants, and SD of daily mean temperature (°C) in the past 3 and 7 days.

### Statistical Analysis

In addition to the influence of contact with infected persons and self-reported ILI status in the past 3 days, as well as age and gender, we first applied logistic regression models to identify health behaviors associated with the probability of self-reported ILI according to the following equation:

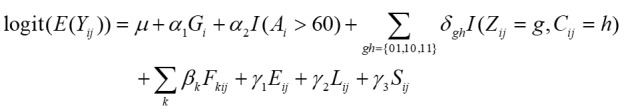



Specifically, we fit the logistic regression model for i=1,2,…,160 and j=1,2,…, D_i_, where G_i_=1 indicates the subject is male, A_i_ is age in years, and D_i_ is the number of diary entries the *i*-th participant provided. The coefficientδ_01_ is one of the relevant parameters representing the effect of contact with infected persons and no self-reported ILI in the past 3 days on risk of having ILI symptoms on the current day.

The explanatory variables were selected into the model in two stages. At the first stage, we considered personal risk factors, including age, gender, contact with persons with ILI or not, average portions of different kinds of foods in the past 3 days or in the past 7 days, staying up late, average number of hours of sleep, sleeping quality, average mood scores, and amount of exercise in the past 3 days or in the past 7 days. The likelihood ratio test was used to select important variables. We then retain the identified influential health behavior variables in the logistic regression models. In the second stage, we continue by identifying influential weather including temperature and humidity and air pollution variables, including PM_2.5_, SO_2_, O_3_, and CO using a stepwise approach. Each variable was computed in two temporal windows. One was for the past 3 days and the other was for the past 7 days. When all potential covariates were fixed in the logistic regression model equation, we further added a random component to the intercept for modeling subject-to-subject variation. Because the influence on the influenza risk of a reported portion of food intake may have been different among the participants, we also added random components to the coefficients of the chosen food items in the regression equation. Finally, we assume that the repeated records of each participant are correlated in the model. All the added random components were assumed to be normally distributed with mean 0 and constant variance. Because each participant provided self-reported ILI status repeatedly during the study period, we further assumed that a pair of responses of a subject had the correlation according to the following equation; 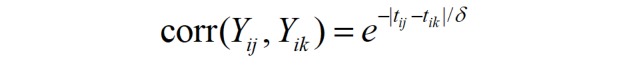
where Y_ij_ is the response of subject *i* on day t_ij_ and δ is a positive parameter to be estimated. We used the R software (R Foundation for Statistical Computing, Vienna, Austria; version 3.3.2) [28] and R package MASS [29] using the glmmPQL function to estimate the parameters in the final mixed-effects logistic regression model.

## Results

### Exploratory Data Analysis

The mean, minimum, and maximum number of days on the participants recorded entries in the health diary were 89, 29, and 183 days, respectively, and for recording the face-to-face contact diary, the same statistics were 91, 6, and 183 during the study period of 183 days, whereas for days with an entry in either diary, the same statistics were 99, 29, and 183 (Table 1). Consequently, we were able to retrieve 14,317 person-days for the health diary and 124,222 face-to-face contacts from the cohort for analysis. The self-reported data from the participants gave an incidence rate estimate of 3.19% (456 ILI persons/14,317 person-days) for the 2015-2016 influenza season. The diary keepers reported 1045 face-to-face contacts with persons definitely having ILI among 124,222 face-to-face contacts, or a chance of 0.84% (1045/124,222), in this influenza season.

**Figure 1:**
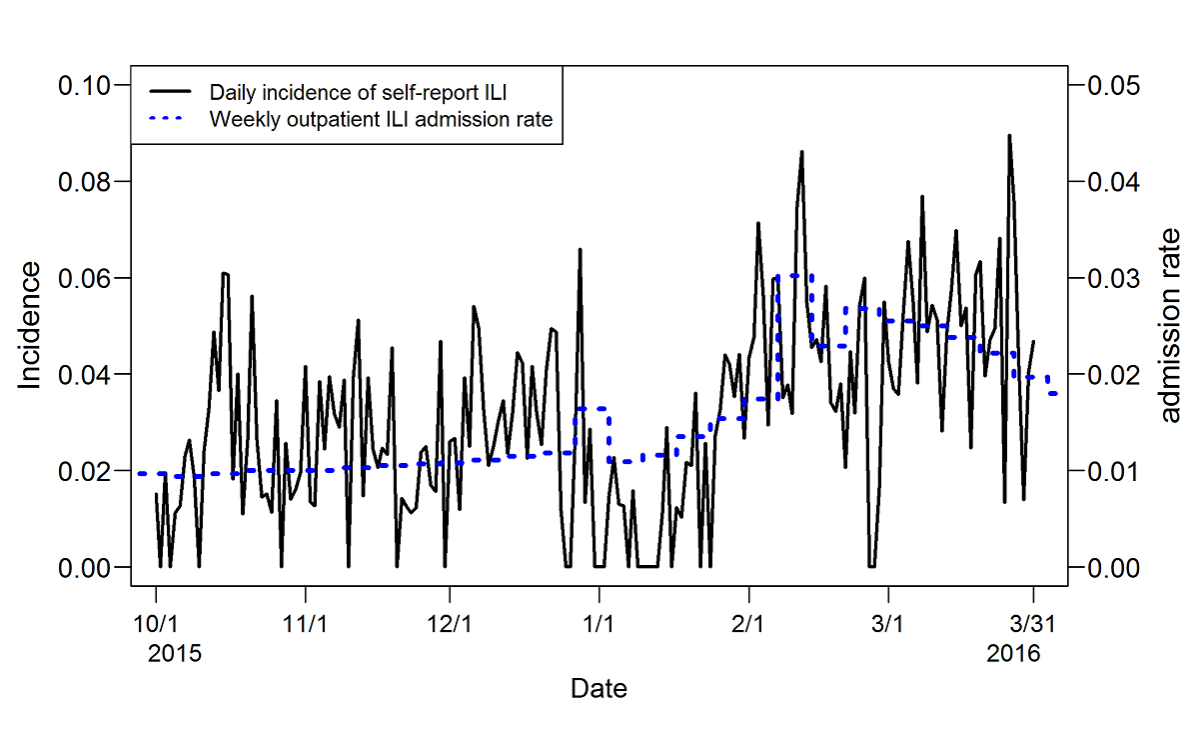
Temporal trends of daily and weekly incidence rate of self-report influenza-like illness (ILI) and weekly outpatient ILI admission rate.

A total of 160 participants were included in this study (Table 2). Most of the participants were female (122/160, 76.3%) and tended to be young adults, aged between 20 and 40 years (99/160, 61.9%). When we considered face-to-face contacts during the previous 3 days, there were 1806 contacts on a given day without any additional contact records from the contact diary for the previous 3 days. Thus, we removed those records from the original 14,317 person-days, and the final total number of person-days was 12,251. Among those contacts (Table 3), for only 131 contacts (131/12,251, 1.07%), both participants and their contacted alters had ILI symptoms during the previous 3 days. For most contacts (10,974/12,251, 89.58%), both participants and their contacted alters were free of ILI. The time series of the daily incidence rate of self-report ILI and the weekly outpatient ILI admission rate plotted in Figure 1 show that the self-reported data of the cohort matched the actual nationwide outpatient surveillance data, especially around the peak, obtained from Taiwan’s Centers for Disease Control. The Pearson correlation was .74 between the weekly incidence rate of self-reported ILI and the weekly outpatient ILI admission rate.

After the first stage of selecting influential variables from using the logistic regression models, there were 12 variables retained in the final model. The descriptive statistics of the identified variables are listed in Table 4. Most of the variables selected here were calculated using the measurements in the prior 3 days, except for the temperature deviation, which used the past 7 days. A total of 12,251 records were included in the final model. The average daily servings of vegetables, fruits, beans and pulses, and dairy products ranged from 0 to 4. The category of cereals comprised two subcategories of food including grains and root vegetables, and rice and noodles, portions of which ranged from 0 to 8 servings. The category of meats and egg contained four subcategories of food, including red meats, white meats, fish, and eggs, which ranged from 0 to 14.5 servings. The proportion of the sleep records in health diaries indicating staying up late (till 1 AM) was 24.96% (3,058/12,251). The average sleeping time was 7.4 hours, with a range from 0.5 to 14.3 hours. The average exercise time was 21 min, ranging from 0 to 120 min. The average temperature deviation in the past 7 days was 2.16°C, ranging from 0.12°C to 5.94°C. The average of maximum PM_2.5_ in the past 3 days was 30 μg/m^3^, ranging from 2 to 88.6 μg/m^3^. The average of maximum 8-hour moving average of O_3_ was 35 ppb, which ranged from 5.7 to 76.2 ppb.

**Table 1:** Descriptive statistics of number of days when participants recorded health diary and face-to-face contact diary, from October 1, 2015 to March 31, 2016.

Type of diary	Number of days					
	Minimum	25%	50%	75%	Maximum	Mean
Health diary	29	63	76	113	183	89
Contact diary	6	64	71	116	183	91
Either diary	29	66	91	128	183	99

**Table 2:** Demographic summary of 160 participants.

Age group	Gender		All
	Male, n (%)	Female, n (%)	n (%)
20-30	14 (36.8)	45 (36.9)	59 (36.9)
31-40	10 (26.3)	30 (24.6)	40 (25.0)
41-50	6 (15.8)	20 (16.4)	26 (16.3)
51-60	2 (5.3)	19 (15.6)	21 (13.1)
61-70	6 (15.8)	8 (6.6)	14 (8.8)
All	38 (100.0)	122 (100.0)	160 (100.0)

**Table 3:** Number and percentage of influenza-like illness (ILI) between participants and their contact persons.

Participants had ILI in past 3 days	Contact persons had ILI in past 3 days	
	No, n (%)	Yes, n (%)
No	10,974 (89.58)	824 (6.72)
Yes	322 (2.63)	131 (1.07)

**Table 4:** Descriptive statistics of selected variables for fitting the models. Q1 and Q3 represent the first and third quartiles.

Variables		Descriptive statistics					
		Minimum	Q1	Median	Mean	Q3	Maximum
**Diet (portion)**							
	Vegetables	0	1	2	1.75	2	4
	Fruits	0	0.5	1	1.28	2	4
	Cereals	0	1.75	2.5	2.55	3	8
	Beans and pulses	0	0	0.5	0.61	1	4
	Meats and eggs	0	1	2	2.34	3.17	14.5
	Dairy products	0	0	0.25	0.4	0.67	4
Sleep time (hours)		0.5	6.5	7.33	7.38	8.25	14.25
Exercise (min)		0	0	15.5	21.44	30.5	120
Temperature deviation (°C)^a^		0.12	1.38	1.92	2.16	2.76	5.94
Maximum PM_2.5_ (μg/m^3^)		2.04	19.25	27.29	30.01	38.02	88.61
Maximum 8-hour moving average of O_3_ (ppb)		5.69	28.17	33.76	34.98	40.78	76.22

^a^Only temperature deviation represented values in the past 7 days; other variables represented values in the past 3 days.

**Table 5:** Estimates of the odd ratios in the mixed-effects logistic regression models. IQR: interquartile range; OR: odds ratio; ILI: influenza-like illness.

Variables		IQR	OR (95% CI)
**Binary variables**			
	Free of ILI and contact with infected persons^a^		1.87 (1.40-2.50)
	Self-reporting ILI and no contact with infected persons^a^		55.79 (45.26-68.77)
	Self-reporting ILI and contact with infected persons^a^		59.97 (44.32-81.14)
	Age >60^b^		0.06 (0.0005-8.23)
	Male^c^		0.30 (0.05-1.76)
	Late bedtime^d^		1.43 (1.11-1.84)
**Continuous variables**			
	Vegetables	1.0	0.92 (0.64-1.33)
	Fruits	1.5	0.37 (0.19-0.75)
	Cereals	1.25	0.99 (0.70-1.40)
	Beans and pulses	1.0	0.42 (0.20-0.87)
	Meats and eggs	2.17	1.09 (0.67-1.77)
	Dairy products	0.67	0.31 (0.14-0.69)
	Sleep duration (h)	1.67	0.97 (0.84-1.12)
	Exercise time	30.5	0.73 (0.63-0.84)
	Temperature deviation	1.37	1.25 (1.13-1.39)
	log (PM_2.5_)^e^	0.68	1.13 (0.99-1.30)
	O_3_	12.66	1.33 (1.20-1.49)
**For two continuous variables**			
	log (PM_2.5_) and O_3_	0.68 and 12.66	1.51 (1.29-1.76)

^a^Reference group: Free of ILI and no contact with infected persons.

^b^Reference group: Age≤60.

^c^Reference group: Female.

^d^Reference group: Did not have late bedtime.

^e^PM_2.5_: fine particulate matter.

### Model Results

The fixed-effects logistic regression models identified several influential variables associated with the probability of participants’ reporting ILI symptoms. The variables include reporting having had any of the following during the past 3 days: contacts with infected persons; being free of ILI; staying up late; average exercise time; and average consumption of fruits, beans and pulses, and dairy products. The most influential environmental variables identified were SD of daily mean temperature in the past 7 days, mean daily maximum 8-hour moving average ozone, and mean daily PM_2.5_ concentrations in the past 3 days. The estimated coefficients of the model shown in Table 5 indicate that the odds ratio (OR) of reporting ILI symptoms was 1.87 with a 95% CI (1.40-2.50) when the participants had contact with persons with ILI symptoms in the past 3 days (hereafter, “recent”). The model estimates indicated that recent consumption of more fruits, beans and pulses, and dairy products significantly decreased the risk of getting ILI. The estimated OR with 95% CI was 0.37 (95% CI 0.19-0.75) for comparing the interquartile range (IQR) of a 1.5-portion increase of daily fruit intake. For recent daily consumption of beans and pulses, the estimated OR was 0.42 (95% CI 0.20-0.87) for comparing an IQR of 1 portion. Comparing an IQR of a two-thirds portion of dairy products, the estimated OR was 0.31 (95% CI 0.14-0.69). Spending more time exercising recently also reduced the risk; the estimated OR was 0.73 (95% CI 0.63-0.84) for comparing an IQR of 30 min. However, staying up later than 1 AM during any night in the past 3 days was associated with increased risk of reporting ILI symptoms, with an estimated OR of 1.43 (95% CI 1.11-1.84).

Variation of daily mean temperature in the past 7 days also increased the risk of infection. The estimated OR was 1.25 (95% CI 1.13-1.39) for comparing an IQR of 1.37°C in SD of the daily temperatures in the past 7 days. Participants exposed to higher ozone concentrations in the past 3 days had a higher chance of reporting ILI symptoms. The estimated OR was 1.33 (95% CI 1.20-1.49) for an increased IQR of 12.7 ppm in the daily maximum 8-hour moving average concentration of ozone. The concentration of PM_2.5_ on a log scale was found to be only marginally associated with response from the fitted model. The estimated OR was 1.13 (95% CI 0.99-1.30) for comparing an IQR of 18.8 μg/m^3^ in daily average concentration of PM_2.5_. Because the two variables of ozone and PM_2.5_ had a correlation of .42, we compared the third quartile concentrations of both ozone and PM_2.5_ against their first quartile levels. The estimated OR increased to 1.5 (95% CI 1.29-1.76). When the participants reported ILI symptoms in the past 3 days, we expected large influence on reporting ILI on the current day even if they had no contact with infected persons. The model estimated OR was 55.8 (95% CI 45.3-68.8).

We removed the insignificant variables of vegetables, cereals, meats and eggs, and sleep time from the model to check whether collinearity among some of the explanatory variables had any influence on the estimated coefficients. The results of the smaller model indicated that the original significance estimates changed only a little. Consumption of vegetables and fruits had a strong correlation of .51. However, we could not find significant association of vegetables with the response when replacing fruits with vegetables even in the smaller model.

### Sensitivity Analysis

We believe that the random effects for modeling participant-to-participant variation and food intake among different people reduced bias of the estimates caused by unobserved factors of the participants. To further examine any selection bias resulting from the fact that the number of weekly entries per participant varied, we conducted one sensitivity analysis to check the robustness of our findings. We randomly selected at most three records per week from each diary keeper to form a subdataset for fitting the same mixed-effects model. The previously mentioned procedure was repeated 100 times to produce 100 sets of estimated coefficients. Then, we calculate the pooled estimate of each coefficient and standard errors of these pooled estimates (Multimedia Appendix 1). The significance levels of pooled estimates of these coefficients were mostly consistent with results of using the whole dataset. We found that only beans and pulses became marginally significant, and staying up late became insignificant.

We also tried another sensitivity analysis to include those participants filling in the diaries less than 10 days per month. By relaxing the inclusion criterion, we included an additional 42 participants in the model. The whole model was then rerun with 202 participants. The results (Multimedia Appendix 2) showed that significant levels of these variable estimates were still consistent with the main finding. The reason for choosing the inclusion criterion of at least 10 days per month (nearly 2-3 days per week) was to consider the short incubation period of influenza. If the participants regularly filled in the diaries, we still can capture the signals at either the early or late stage of an ILI episode.

## Discussion

### Principal Findings

The online diary-based approach in this study was used to collect not only ILI symptoms but also daily health behaviors and participants’ social networks. This innovative approach can reduce recall bias compared with weekly or monthly surveys. Although the presence of significant risk factors for ILI has been revealed in different studies, few have quantitatively estimated the risk levels associated with different factors. In this study, we had the opportunity to collect all those risk factors from online diaries during an influenza season. We have used these empirical observations to reveal several risk reduction–related health behaviors such as avoiding contact with persons with ILI, sleeping earlier, keeping a good diet, exercising more, and being aware of environmental temperature and air pollution. Our study demonstrated a wide spectrum of ILI risks at the personal, contact, and environmental levels.

In traditional approaches to studying interpersonal influenza transmission, the main focus was identifying people with ILI symptoms or confirmed ILI in a hospital setting and then following up with the potential transmission within the household or in schools [10,30]. One study with a flu watch cohort in the United Kingdom also tried to recruit healthy participants from volunteers among general practitioners to monitor the influenza activity, severity, and virus evolution in the community [31]. The participants needed to record their contact patterns and activities before and during illness. However, these ILI symptoms were only collected from the ego. Without the ILI status of their contacted alters, we cannot observe influenza transmission. Similarly, in our study, we have self-reported ILI status of the diary keepers at least 2 to 3 days a week during the study period. ILI status of the alters was recorded only when they were contacted by the diary keepers, and this information was thus incomplete. The limited data create a hurdle for examining the dynamics of influenza transmission among alters in the egocentric networks although we can estimate the influence of alters on egos.

The household cohort can provide insights to capture the dynamics of influenza transmission after identifying an index case in a household [10]. It requires substantial resources to confirm the infected cases and closely follow up on the series of infections. The transmission setting focused on the household and family members. In this study, we tried to understand not only ILI transmission risk in the household but also in other settings where the participants had contact with others. In our online diary, the relationship between the participant and the person with whom they had contact and the place where they had contact were all recorded. Therefore, we did further stratification analysis for different relationships of contact. We stratified our contacts into two categories, contact with family members and contact with other people, to refit the mixed-effects model. The results showed that participants reporting contact with nonrelatives had higher risk of getting ILI than those who had contact with family members (Multimedia Appendix 3). It might be that participants knew of their family members’ ILI symptoms early, so they were able to take some preventive measures. The other reason might be that more than 70% of our participants were college students and young adults who had high chances of close contact with classmates, good friends, and coworkers.

In this study, we were able to quantify the risk of developing ILI from having been in contact with persons with ILI in the past 3 days. The estimated OR of 1.87 in our model is quite similar to a household transmission study conducted in France, which also reported that the hazard ratio (HR) of increased risk of influenza transmission in preschool contacts was 1.85 compared with school age and adult contacts [32]. They also reported an increased risk in contacts exposed to preschool index patients (HR=1.93) and school-age index patients (HR=1.68), compared with those exposed to adult index cases.

We also found that having a later bedtime in the past 3 days is a significant risk factor for developing ILI. In children, staying up late has previously been found to be associated with poorer quality of life and overall health [33]. Although sleep duration and quality of sleep were not significantly associated with the chance of getting ILI in our study, later bedtime may serve as a proxy measure of sleeping quality. Good sleep is correlated with good immunity function, especially for the primary response to infection. One study in the United States recruited 153 healthy volunteers to observe the association between sleep duration and sleep efficiency and development of a clinical cold over 14 days [34]. Their results showed that participants with less than 7 hours of sleep were 2.94 times as likely (95% CI 1.18-7.30) to develop a cold than those with 8 or more hours of sleep. Poor sleep efficiency was also a very strong risk factor for developing a cold (OR 5.50, 95% CI 2.08-14.48) [34].

Diet and physical exercise are also very important health behaviors to enhance immunity and reduce the chance of influenza infection. Although we have considered many types of foods in the model, we finally found that fruits, beans and pulses, and dairy products are associated with lower ILI risk. In the literature, one study found that anthocyanins from fruit extracts inhibited influenza virus adsorption into cells and also virus release from infected cells [35]. One cohort study over 12 influenza seasons in Ontario, Canada, found that compared with inactive individuals, moderately active (OR 0.83, 95% CI 0.74-0.94) and active (OR 0.87; 95% CI 0.77-0.98) individuals were less likely to make an influenza-coded visit [36]. For those individuals aged less than 65 years, the protective effects of activity are still significant (active OR 0.86, 95% CI 0.75-0.98, moderately active: OR 0.85, 95% CI 0.74-0.97). Our result also showed that 30 min of exercise time was associated with a lower chance of getting ILI (OR 0.76, 95% CI 0.65-0.89).

In addition to personal risk factors, environmental factors such as temperature variation, PM_2.5_, and O_3_ exposure are also associated with immunity, virus transmission, and replication from the literature. In one guinea pig study, cold and dry conditions were best for influenza transmission between hosts [37]. Previous studies have focused more on how seasonal temperature variation affected influenza epidemics. One study showed that a 1°C decrease in temperature would increase the risk of influenza infection by 11% (OR 1.11, 95% CI 1.03-1.20) [38]. Although most studies did not find an effect of temperature variation on influenza activity, our results from this unique dataset imply that this relationship is worth further investigation.

Positive correlations between PM_2.5_ and ILI have been found in many studies in Beijing, China [39,40]. They found that the effect on influenza incidence caused by PM_2.5_ had an increasing gradient as PM_2.5_ increased when PM_2.5_ concentration was larger than 70 μg/m^3^. Another study involving 47 Chinese cities also showed that the effect of ambient PM_2.5_ on influenza incidence occurred at lag 2 day, with a relative risk of 1.015 (95% CI 1.004-1.025) associated with a 10μg/m^3^ increase in PM_2.5_ [15]. In addition to epidemiological findings showing a connection between ILI and PM_2.5_, one study conducted in Taiwan isolated influenza virus and avian influenza virus from ambient samples during Asian dust storm days [41]. A possible mechanism is that influenza virus was carried by airborne particles and then inhaled into the respiratory tract. That study also found that ILI risk only increased with elevated PM_2.5_ during flu season and was not significant during nonflu season. Another air pollutant, O_3_, was also found to induce cleavage of the influenza A hemagglutinin (HA) protein, and the secreted endogenous proteases were sufficient to activate HA, leading to a significant increase in viral replication [16].

One social network study [12] found that people with more social ties might have higher chances of getting infection or spreading one to others. In our study, we also included the average number of contact persons in the past 3 days in the model, but the results did not show statistical significance. One nationwide survey in Taiwan found that people with a higher number of contacts also had a higher likelihood of getting an influenza vaccination [42]. Thus, people with more social ties will also take some preventative measures against influenza; that might be the reason we could not find a correlation between ILI and the number of social ties in this study.

### Limitations

There are several limitations in this study. The first one is a lack of laboratory samples to confirm whether the participants or the contact alters were in fact influenza infected or not. From a practical viewpoint, it is not possible to know who transmitted the influenza virus among the general public. Thus, we treat this uncertainty as a random effect for each participant. Furthermore, we used a relatively strict definition of ILI in our questionnaire. We treated participants as getting ILI only when they selected “definitely having ILI symptoms” and clicked specific symptoms in the symptom list. Using this definition, our data showed a good match with the epi-curve of ILI from the nationwide outpatient surveillance. The second limitation is the sample size and representativeness. Because of the nature of our study design, the diary-based follow-up needs patience and persistence to record the health and contact diary for 6 months. It is difficult to keep a large number of participants in a study of this kind for such a long time. Also, volunteer-based online surveys can never claim to be representative of the general population. Due to the limitation of small sample size, we cannot generalize our findings to the general population. In particular, we have found that the participants in this study tended to be young, and there was a large proportion of female participants (76.3%, 122/160). Therefore, we included age and sex in the model for adjustment. In addition, we also incorporated other variables such as geographic area into the model, with no significant difference.

The third limitation is lack of data on protective behaviors such as wearing a facial mask and vaccination status during the study period. In fact, we collected the vaccination status before and after influenza season. However, we did not know the exact date of vaccination. Therefore, it was not available to measure the effect of this diary-based study design. The fourth limitation is lack of exercise intensity in this diary. We only asked participants to record the duration of exercise. From the current findings, the exercise time should be longer than 30 min to show reduced risk of getting ILI. The fifth limitation is related to exposure estimation. In fact, we did not know exactly where the participants were located. They only reported the township where they lived. Therefore, we were only able to use a station in the corresponding township or the closest station to it. The concentrations of PM_2.5_ and ozone could be treated only as environmental exposure, not as a measure of personal exposure.

### Conclusions

In conclusion, our study shows that keeping a healthier lifestyle, including having a nutritious diet, sleeping earlier, and doing longer physical exercise is associated with a lower risk of getting ILI. Self-protection and avoiding contact with infected persons, as well as keeping alert to temperature changes and air quality are also linked to lower risk of getting ILI.

## Appendix

Multimedia Appendix 1 Comparison of estimated coefficients using the whole dataset and pooled estimates of 100 sampled subdatasets.

Multimedia Appendix 2 Sensitivity analysis on model estimation by including those participants filling in the diaries less than 10 days per month.

Multimedia Appendix 3 Estimation of ILI risk by stratifying two types of contacts with infected family members and nonrelatives.

## Abbreviations

ILI: influenza-like illness
HR: hazard ratio
IQR: interquartile range
OR: odds ratio
PM2.5: particulate matter
