# Supplementary material for: Estimating the Risk of Influenza-Like Illness Transmission Through Social Contacts: Web-Based Participatory Cohort Study
Source: JMIR Public Health Surveill. 2018 Apr 9;4(2):e40. doi: 10.2196/publichealth.8874 (PMC5913573; doi:10.2196/publichealth.8874)
Supplement: Multimedia Appendix 1 [file publichealth_v4i2e40_app1.pdf]

Appendix 1. Comparison of estimated coefficients using the whole dataset and pooled estimates of 100 sampled sub-datasets

	Whole dataset			100 sub-datasets		
Variables	Estimate	S.E.	p-value	Estimate	S.E.	p-value
Intercept	-6.6116	0.6602	0.0000	-6.4246	0.8211	0.0000
Free of ILI and contact with infected persons	0.6269	0.1478	0.0000	0.6119	0.2396	0.0107
Self-reporting ILI and no contact with infected persons	4.0216	0.1067	0.0000	4.2034	0.1623	0.0000
Self-reporting ILI and contact with infected persons	4.0938	0.1543	0.0000	4.3143	0.2504	0.0000
Age > 60	-2.7858	2.4965	0.2662	-2.4635	2.3381	0.2937
Male	-1.2057	0.9024	0.1835	-1.2276	0.8383	0.1451
Vegetables	-0.0796	0.1850	0.6670	-0.0564	0.2007	0.7786
Fruits	-0.6590	0.2367	0.0054	-0.6722	0.2455	0.0062
Cereals	-0.0074	0.1410	0.9581	-0.0079	0.1396	0.9551
Beans and pulses	-0.8632	0.3688	0.0193	-0.6696	0.3642	0.0660
Meats and eggs	0.0394	0.1139	0.7293	0.0155	0.1166	0.8943
Dairy products	-1.7568	0.6126	0.0041	-1.6769	0.6214	0.0070
Late bedtime	0.3557	0.1304	0.0064	0.2860	0.1971	0.1469
Sleep duration (hours)	-0.0185	0.0454	0.6841	-0.0139	0.0603	0.8178
Exercise time	-0.0104	0.0025	0.0000	-0.0106	0.0035	0.0023
Temperature deviation	0.1639	0.0396	0.0000	0.1638	0.0522	0.0017
log ($PM_{2.5}$)	0.1801	0.1026	0.0792	0.1557	0.1394	0.2641
O_3	0.0228	0.0044	0.0000	0.0243	0.0058	0.0000
