# Supplementary material for: Estimating the Risk of Influenza-Like Illness Transmission Through Social Contacts: Web-Based Participatory Cohort Study
Source: JMIR Public Health Surveill. 2018 Apr 9;4(2):e40. doi: 10.2196/publichealth.8874 (PMC5913573; doi:10.2196/publichealth.8874)
Supplement: Multimedia Appendix 2 [file publichealth_v4i2e40_app2.pdf]

Appendix 2. Sensitivity analysis on model estimation by including those participants filling in the diaries less than 10 days per month

Variables	Reference group or IQR	OR (95%C.I.)
**Binary variables:**		
Free of infection and contact with infected	Free of ILI and no contact with ILI	1.47 (1.12, 1.94)
Self-reported infection and no contact with infected	Free of ILI and no contact with ILI	47.42 (39.19, 57.38)
Self-reported infection and contact with infected	Free of ILI and no contact with ILI	48.93 (37.14, 64.45)
Age > 60	Age <=60	0.06 (0.00, 9.21)
Male	Female	0.39 (0.08, 2.01)
Late bedtime	Did not sleep late	1.34 (1.06, 1.69)
**Continuous variables:**		
Vegetables	IQR=1.0	0.90 (0.64, 1.27)
Fruits	IQR=1.5	0.43 (0.23, 0.80)
Cereals	IQR=1.25	0.90 (0.63, 1.28)
Beans and pulses	IQR=1.0	0.34 (0.15, 0.78)
Meats and eggs	IQR=2.17	1.02 (0.66, 1.59)
Dairy products	IQR=0.67	0.27 (0.13, 0.57)
Sleep duration (hours)	IQR=1.67	0.98 (0.86, 1.12)
Exercise time	IQR=30.5	0.83 (0.73, 0.94)
Temperature deviation	IQR=1.38	1.25 (1.13, 1.38)
log ($PM_{2.5}$)	IQR=0.69	1.10 (0.97, 1.25)
O_3	IQR=12.68	1.31 (1.19, 1.45)
**Two continuous variables:**		
log ($PM_{2.5}$) and O_3	IQR=(0.69, 12.68)	1.44 (1.25, 1.67)

IQR: Interquartile range; N=202 participants
