# Supplementary material for: Estimating the Risk of Influenza-Like Illness Transmission Through Social Contacts: Web-Based Participatory Cohort Study
Source: JMIR Public Health Surveill. 2018 Apr 9;4(2):e40. doi: 10.2196/publichealth.8874 (PMC5913573; doi:10.2196/publichealth.8874)
Supplement: Multimedia Appendix 3 [file publichealth_v4i2e40_app3.pdf]

Appendix 3. Estimation of ILI risk by stratifying two types of contacts with infected family members and non-relatives

Variables	Reference group or IQR	OR (95%C.I.)
**Binary variables:**		
Self-reporting ILI and no contact with infected	Free of ILI and no contact with infected	51.47 (41.90, 63.23)
Free of ILI and contact with:		
infected non-relatives	Free of ILI and no contact with infected	1.97 (1.39, 2.78)
infected family members	Free of ILI and no contact with infected	1.30 (0.88, 1.92)
Self-reporting ILI and contact with:		
infected non-relatives	Free of ILI and no contact with infected	56.89 (39.75, 81.40)
infected family members	Free of ILI and no contact with infected	13.18 (8.73, 19.91)
Age > 60	Age <=60	0.06 (0.00, 11.22)
Male	Female	0.35 (0.05, 2.24)
Late bedtime	Did not sleep late	1.46 (1.13, 1.88)
**For a continuous variable:**		
Vegetables	IQR=1.0	0.85 (0.57, 1.27)
Fruits	IQR=1.5	0.39 (0.19, 0.79)
Cereals	IQR=1.25	0.99 (0.69, 1.42)
Beans and pulses	IQR=1.0	0.35 (0.16, 0.78)
Meats and eggs	IQR=2.17	1.03 (0.62, 1.71)
Dairy products	IQR=0.67	0.31 (0.13, 0.70)
Sleep duration (hours)	IQR=1.67	0.98 (0.85, 1.14)
Exercise time	IQR=30.5	0.72 (0.62, 0.84)
Temperature deviation	IQR=1.37	1.24 (1.11, 1.38)
log ($PM_{2.5}$)	IQR=0.68	1.14 (0.99, 1.30)
O_3	IQR=12.66	1.30 (1.17, 1.45)
**For two continuous variables:**		
log ($PM_{2.5}$) and O_3	IQR=0.68 and 12.66	1.48 (1.27, 1.73)

IQR: Interquartile range
